# Betaine enhances the cellular survival via mitochondrial fusion and fission factors, MFN2 and DRP1

**DOI:** 10.1080/19768354.2018.1512523

**Published:** 2018-08-30

**Authors:** Min Jung Kim

**Affiliations:** Department of Biological Sciences, Sookmyung Women’s University, Seoul, South Korea

**Keywords:** Betaine, mitochondrial dynamics, fusion, fission

## Abstract

Betaine is a key metabolite of the methionine cycle and known for attenuating alcoholic steatosis in the liver. Recent studies have focused on the protection effect of betaine in mitochondrial regulation through the enhanced oxidative phosphorylation system. However, the mechanisms of its beneficial effects have not been clearly identified yet. Mitochondrial dynamics is important for the maintenance of functional mitochondria and cell homeostasis. A defective mitochondrial dynamics and oxidative phosphorylation system have been closely linked to several pathologies, raising the possibility that novel drugs targeting mitochondrial dynamics may present a therapeutic potential to restore the cellular homeostasis. In this study, we investigated betaine’s effect on mitochondrial morphology and physiology and demonstrated that betaine enhances mitochondrial function by increasing mitochondrial fusion and improves cell survival. Furthermore, it rescued the unbalance of the mitochondrial dynamics from mitochondrial oxidative phosphorylation dysfunction induced by oligomycin and rotenone. The elongation properties by betaine were accompanied by lowering DRP1 and increasing MFN2 expression. These data suggest that betaine could play an important role in remodeling mitochondrial dynamics to enhance mitochondrial function and cell viability.

## GRAPHICAL ABSTRACT

**Figure:**
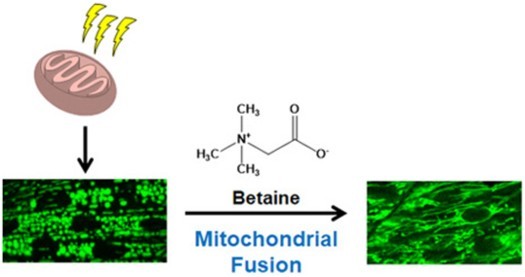

## Introduction

Mitochondria are essential organelles for providing the majority of cellular energy ATP through oxidative phosphorylation (OXPHOS), maintaining homeostasis, oxygen metabolism, cell survival and apoptosis (Friedman and Nunnari 2014). Mitochondrial OXPHOS dysfunction has been reported to many detrimental effects on cardiovascular disease, neurodegenerative disease and cancer (Picard et al. 2016).

Mitochondria are dynamic networks that constantly undergo fusion and fission (Suarez-Rivero et al. 2016). These processes lead to the quality control of mitochondria to determine their survival. A fused mitochondrion is related to a higher ATP synthesis to maintain homeostasis, which is essential for cell survival. Whereas fragmented mitochondria display a reduced respiration and early signs of apoptosis in cancerous cells. The mitochondrial morphology is tightly connected to mitochondria-related pathologies. Many human diseases like MILS (Maternally Inherited Leigh Syndrome), CMT2A (Charcot-Marie-Tooth disease type 2A), Parkinson and Alzheimer are characterized by an alteration of mitochondrial dynamics (Chauhan et al. 2014).

Mitochondrial division is mediated by dynamin related protein 1 (DRP1) which facilitates fission to activate apoptosis and mitofusins (MFNs) which mediates fusion to have an anti-apoptotic effect (Westermann 2012). CMT2A has been characterized by suppression of mitochondrial fusion from MFN2 mutation and MFN2 agonists promoted the fusion and rescue the dominant mitochondrial defects through normally redistributing abnormal axonal mitochondrial trafficking in the nerves of CMT2A mice (Rocha et al. 2018). Since therapeutics directly targeting mitochondrial dynamics do not exist so far, the MFN2 agonists reveal a promising therapeutic approach that enhances mitochondrial fusion or trafficking.

Betaine (N,N,N-trimethylglycine) is an essential nutrient that has been studied for cytoprotective properties such as an osmolyte in cell volume maintenance and correction of a defective cellular methylation against environmental stress (Day and Kempson 2016). The beneficial therapeutic effects of betaine have been shown: the decrease of plasma homocysteine concentration in hyperhomocysteinemia related to atherosclerotic disease (Selicharova et al. 2013), the attenuation of hepatosteatosis and steatohepatitis from an alcoholic, and nonalcoholic liver damage (Houghton 2003). Moreover, pretreatment of betaine shows potential as a cytoprotective agent (Abdelmalek et al. 2001). It is highly effective to protect liver damage by toxins like chloroform and LiCl, reduce neurotoxicity against rotenone, and strengthen hepatocytes mitochondria function from ethanol-induced models and alleviate cellular damage like blunting ROS (Kharbanda et al. 2005; Kharbanda et al. 2009) and enhancing mitochondrial respiration to the mitochondrial OXPHOS and oxidative stress (Lee 2015). Although the cytoprotective properties of betaine have been studied, the mechanisms of betaine over mitochondrial dysfunctions remains unclear.

More data have been pointed out the essential role of betaine in the preservation of mitochondrial functions. One purpose here is to identify the effects of betaine on mitochondrial dysfunctions. We showed that betaine treatment can affect the mitochondrial dynamics by increasing mitochondrial fusion to enhance mitochondrial function using Huh7, human hepatocarcimona cells. The effect of betaine was further expanded when the mitochondria were challenged by inhibitors, oligomycin and rotenone in Huh7 and zebrafish embryo. Our results not only demonstrated that betaine restored the mitochondria fragmentation induced by oligomycin or rotenone but also discovered a cytoprotective mechanism of betaine that enhances cell survival and mitochondrial membrane potential.

## Materials and methods

### General reagents

Betaine (Cat. No. B2629) and other chemicals were purchased from Sigma-Aldrich unless otherwise specified.

### Cell culture and transfection

Human HCC (Huh7) cells were transfected with the dsRed2-MLS vectors using the Turbofect^TM^ transfection reagent. After 24 hours of transfection, cells were pretreated with or without mitochondrial inhibitors for 2 hours and then co-incubated with betaine for additional 24 hours.

### Zebrafish care and maintenance

All zebrafish (*Danio Rerio*) husbandry and experimental protocols complied with institutional guidelines were approved by local ethics boards (Sookmyung Women’s University Animal Care and Use Committee, SMWU-IACUC-1712-036) (Westerfield 2000). Zebrafish were maintained under standard conditions and embryos were obtained from natural crosses between Tg(*mito:*EGFP) zebrafish (Kim et al. 2008).

### Confocal live imaging and measurement of mitochondrial length of Huh7 cells and zebrafish embryos

For mitochondria imaging of Huh7 cells, dsRed2-MLS transfected Huh7 cells were seeded onto 0.1% poly-D-lysine-coated coverslips and cultured for 24 hours. For the live imaging of mitochondria in zebrafish embryos, embryos at 1 dpf (day post fertilization) were pretreated with 2 μM oligomycin for 15 minutes and then co-incubated with betaine for 6 hours at 28.5°C. Tg(*mito*:EGFP) zebrafish embryos were anesthetized in 0.02% tricaine, and mounted with 3% methylcellulose. Z-series of images (10 images; interval thickness: 1 μm) were collected with an LSM 700 confocal laser microscope (Carl Zeiss) and presented as a stacked image. The mitochondrial length was analyzed as average lengths from 10 mitochondria of individual cells using ImageJ (NIH, USA).

### Determination of mitochondrial membrane potential in Huh7 cells

Cells were pretreated with mitochondrial inhibitors for 2 hours, then betaine was added for 24 hours following incubation with 200nM MitoStatus TMRE and Hoechst 33342 (5μg/mL) for 30 min. Images were taken using an LSM710 confocal microscope. The signals were examined using identical settings to obtain the same sensitivity.

### Cell viability assay

Cell viability was assessed with MTT. Absorbance was measured at 550 nm with a SpectraMax® M5 microplate reader.

### Western blotting

Whole-cell lysates (30 μg) were loaded on 10–15% SDS-PAGE and incubated with primary antibodies against β-actin, DRP1, GFP and MFN2 (Santa Cruz), and then incubated with the HRP-conjugated secondary antibody (Thermo Scientific). Signals were detected with SuperSignal West Pico Chemiluminescent Substrate (Thermo Scientific) using LI-COR (LI-COR).

### Statistical analysis

The data are presented as the mean and standard deviation of the results from three independent experiments (*n* = 3). The statistical significance of the experimental differences was determined with two-way analysis of variance. *P* values less than .05 were considered statistically significant, and significance is indicated on the graphs with asterisks.

## Results

### Betaine promotes mitochondrial fusion in Huh7 cells

**Figure 1.**
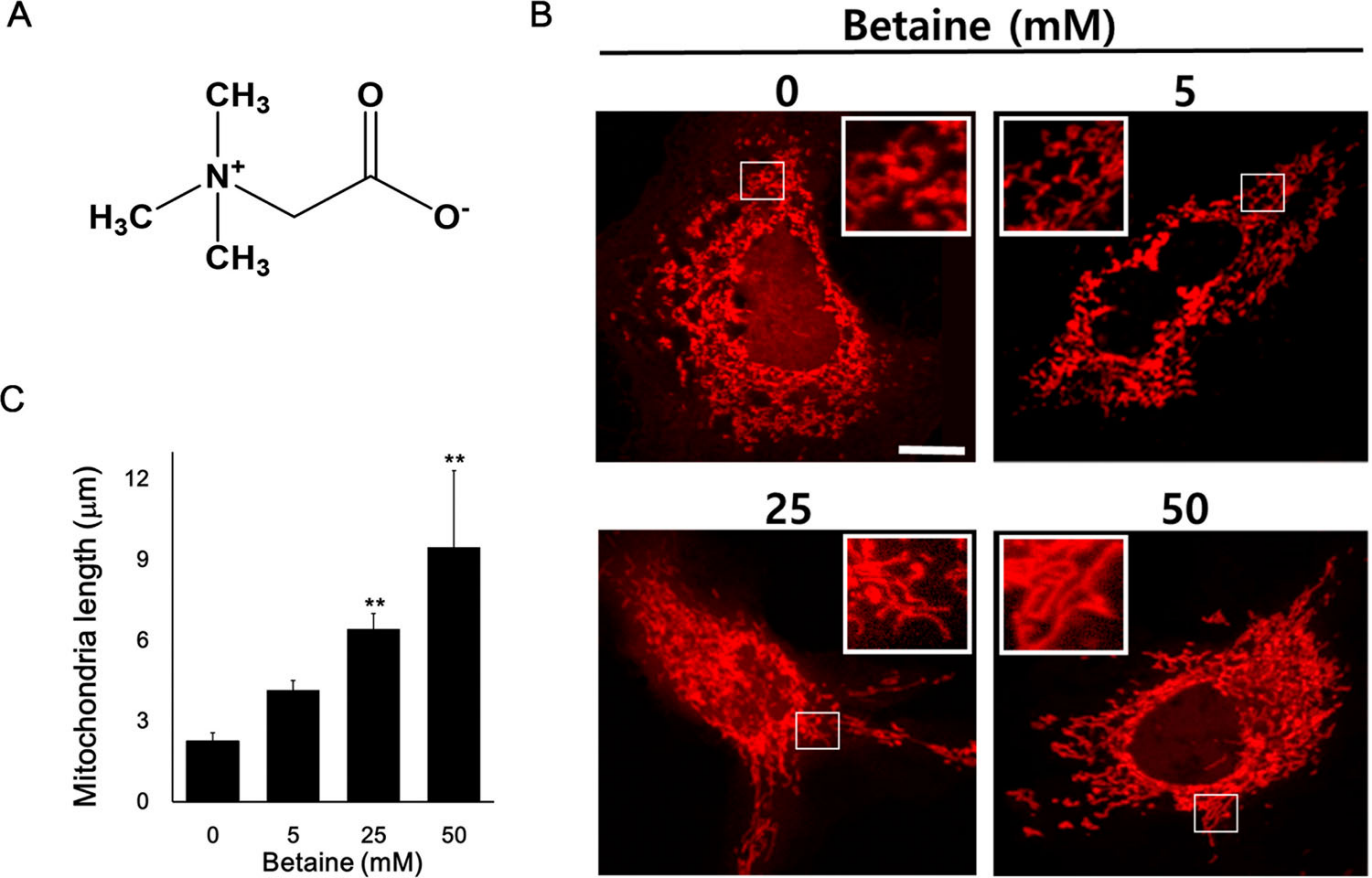
Structure of betaine and effect of betaine on mitochondrial fusion of Huh7 cells. (A) Structure of betaine. (B) Representative images of mitochondria-targeted dsRed showing the effect of betaine on mitochondrial fusion. Scale bar = 5 μm. Insets represent magnification of the boxed area. (C) Quantitative analysis of mitochondrial length. Data shown are the means ± SEM of measurements taken from 100 individual cells from 3 independents experiments. **p < .001 compared to 0 mM betaine.

Betaine is a methyl derivative of the amino acid glycine with a molecular formula of (CH_3_)_3_N^+^CH_2_COO^−^ and known as a methylamine (Day and Kempson 2016) (Figure 1(A)). To evaluate betaine effects on mitochondrial dynamics, we used Huh7 cells expressing mitochondria-targeted dsRed to perform a live imaging experiment for mitochondrial change. Cells were incubated with betaines for 24 hours and the length and morphology of mitochondria were analyzed to determine the change in mitochondrial dynamics (Figure 1). When most of control cells had normal size of mitochondria (average length about 2.2 μm), strikingly, cells with the different concentrations (0, 5, 25 and 50 mM) of betaine treatment had the increased average length of mitochondria in a dose-dependent manner (2.26, 4.1, 6.4 and 9.5 μm respectively, Figure 1(C)). Cells with 50 mM betaine contained ∼ 4 times longer mitochondria than control cells. Also, the results indicated that betaine changed the morphology of mitochondria. While control cells without betaine showed small and tubular-shaped mitochondria whereas with higher concentrations of betaine tended to be elongated and hyper-fused (Figure 1(B). insets show small and tubular-shaped mitochondria for 0 mM betaine to large and hyper-connected ones for 50 mM).

### Betaine improves mitochondrial dynamics from OXPHOS dysfunction

**Figure 2.**
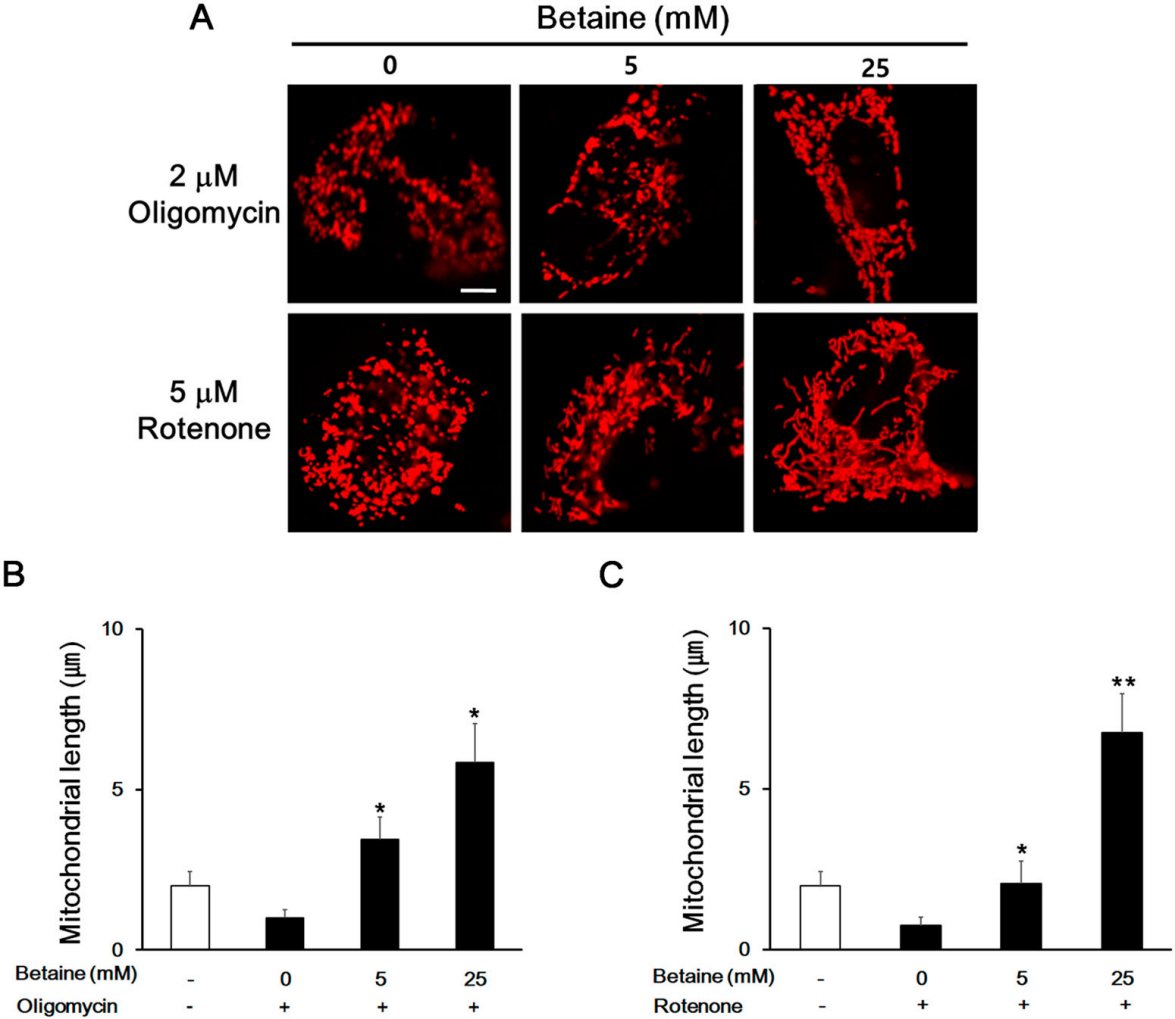
The effect of betaine on mitochondrial dynamics from the OXPHOS dysfunction. (A) Representative images showing the mitochondrial fusion effect of betaine in the presence of mitochondrial inhibitors. Scale bar = 5 μm. (B and C) Quantitative analysis of mitochondrial length. Treatment of betaine prevented oligomycin- (B) or rotenone- induced (C) mitochondrial fragmentation. Data shown are the means ±SEM of measurements taken from 100 individual cells from 3 independents experiments. *p < .05 and **p < .001 compared to 0 mM betaine with inhibitor.

Considering that the fragmentation of mitochondria is the early phenotype of apoptosis, we investigated if betaine can restore mitochondrial fragmentation caused by oxidative phosphorylation (OXPHOS) dysfunction. We used mitochondrial inhibitors for disruption of mitochondrial respiratory chain function through inhibition of electron transport at Complex I (rotenone) or blockage of ATP synthesis through inhibition of ATP synthase (oligomycin) (Byrnes et al. 2018). The concentrations of inhibitors were determined not to cause irreversible damage to the mitochondrial shape. Oligomycin or rotenone treatment changed the mitochondrial length from ∼ 2.2 μm to 0.75 μm with the round- fragmented mitochondria (Figure 2). Interestingly, these round-fragmented morphology induced by the treatment of oligomycin or rotenone was nearly returned to the normal shape with betaine. Moreover, the average length of mitochondria with 25 mM betaine after inhibitor challenges recovered almost to the basal levels of with 25 mM betaine without inhibitor challenges. These results indicate that betaine can promote the mitochondrial fusion as well as restore the mitochondrial dynamics from mitochondrial OXPHOS dysfunction.

### Betaine regulates expression of mitochondrial fusion/fission factors

**Figure 3.**
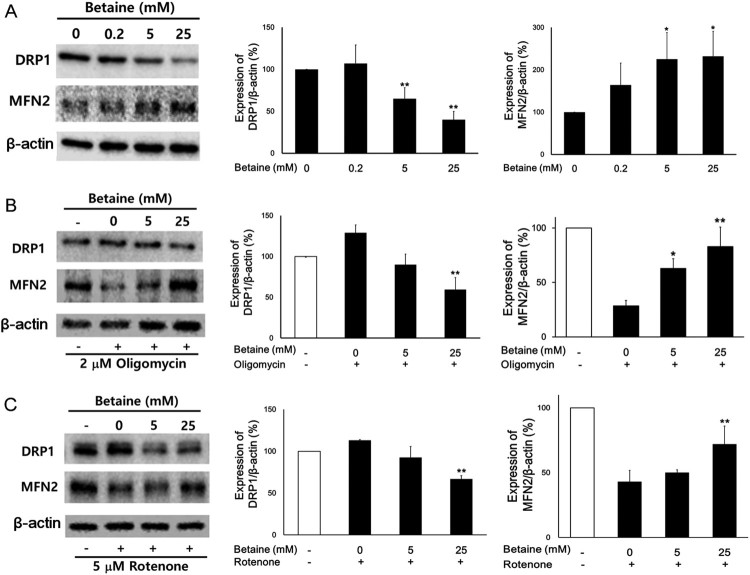
Effects of betaine on expressions of mitochondrial fusion and fission related proteins. (A) Cells with betaine treatment for 24 hours were lysed for Western blot analysis. Huh7 cells were pretreated with 2 μM oligomycin (B) or 5 μM rotenone (C) and co-incubated with betaine Quantitative analysis of relative intensity of DRP1 and MFN2 are presented. Data shown are the means ± SEM of measurements taken from three independent experiments. *p < .05, **p < .001 compared to 0 mM betaine (A) and 0 mM betaine with inhibitor (B and C).

Our findings about the reliable effect of betaine on the mitochondrial morphology raise the possibility that betaine-mediated change of mitochondrial morphology might be mediated by the mitochondrial fission-fusion events. Since the levels of DRP1 and MFN2 control the mitochondrial fission and fusion activities respectively (Zamponi et al. 2018), we examined the expression levels of these two proteins (Figure 3). The level of DRP1 was significantly decreased in response to betaine in a dose-dependent manner. The 25 mM betaine treatment induced nearly 60% decrease in DRP1 expression. In contrast to the reduction of DRP1 expression by betaine, MFN2 was overexpressed more than 2.5 times by betaine treatment. These data suggest that betaine increases the expression levels of MFN2 enhancing the mitochondrial fusion process and decreases the levels of DRP1 suppressing the fission mechanism of mitochondrial dynamics.

To investigate whether betaine modulates mitochondrial length and shape through DRP1 and MFN2 proteins on the dysfunctional mitochondria, we tested the levels of DRP1 and MFN2 of oligomycin or rotenone challenged cells. The levels of DRP1 were ∼30% and ∼15% increased with oligomycin and rotenone, respectively by comparing to those of cells without inhibitor treatment. As shown in Figure 3(B,C), the levels of DRP1 were deceased by betaine treatment compared to oligomycin or rotenone treated cells. Higher concentrations of betaine caused a strong reduction by 50% in DRP1 expression. On the other hand, the levels of MFN2 were significantly decreased by 70% and 55% from oligomycin and rotenone treatments, respectively. 25 mM betaine increased MFN2 expression by 150% after oligomycin challenge while by 59% after rotenone. The effects of betaine on DRP1 and MFN2 expressions after disruption of mitochondrial OXPHOS function by oligomycin or rotenone were increased in a dose-dependent manner. However, the recovery of MFN2 was more profound than DRP1 in oligomycin treated cells. Taken together, these data imply that betaine regulates the protein levels of DRP1 and MFN2 resulting in mitochondrial remodeling.

### Betaine augments cell survival and enhances mitochondrial membrane potential

**Figure 4.**
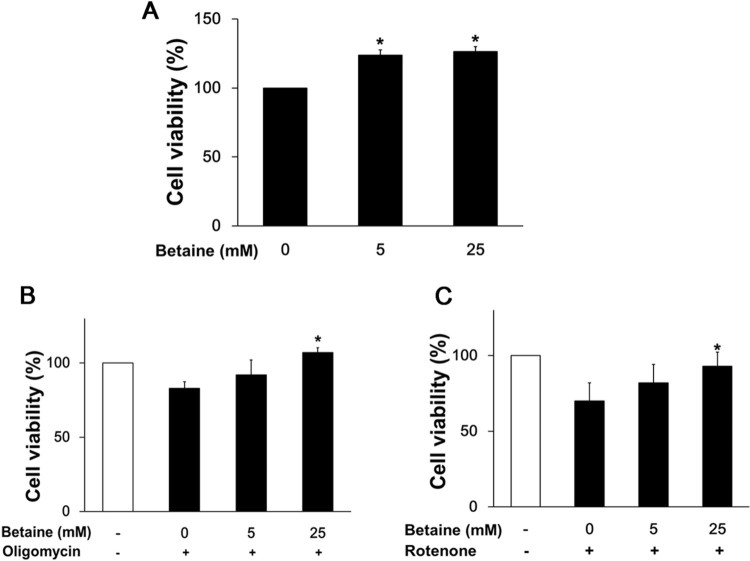
Effect of betaine on the survival of Huh7 cells. (A) After 24 hours treatment of betaine, cells were subjected to MTT assay. Two μM oligomycin (B) or 5 μM rotenone (C) were used to pretreat and co-incubated with betaine to explore the effect of betaine over inhibitor-induced OXPHOS dysfunction. Data are means ± SEM of triple independent experiments. *p < .05 vs 0 mM betaine (A) and 0 mM betaine with mitochondrial inhibitor (B and C).

To explore the functional impact of betaine on cell survival, we examined the change in cell viability by betaine treatment using the MTT assays. Betaine robustly increased 20% cell viability over control cells (Figure 4). Since betaine treatment which caused the elongated mitochondrial phenotype improved cell survival, we tested whether it affects cell survival against OXPHOS dysfunction induced by oligomycin or rotenone. The cell viability was reduced to 15% and 30% by oligomycin and rotenone, respectively compared to the control cells. The recovery of cell viability from mitochondrial dysfunction corresponded with an increase of betaine concentrations.

**Figure 5.**
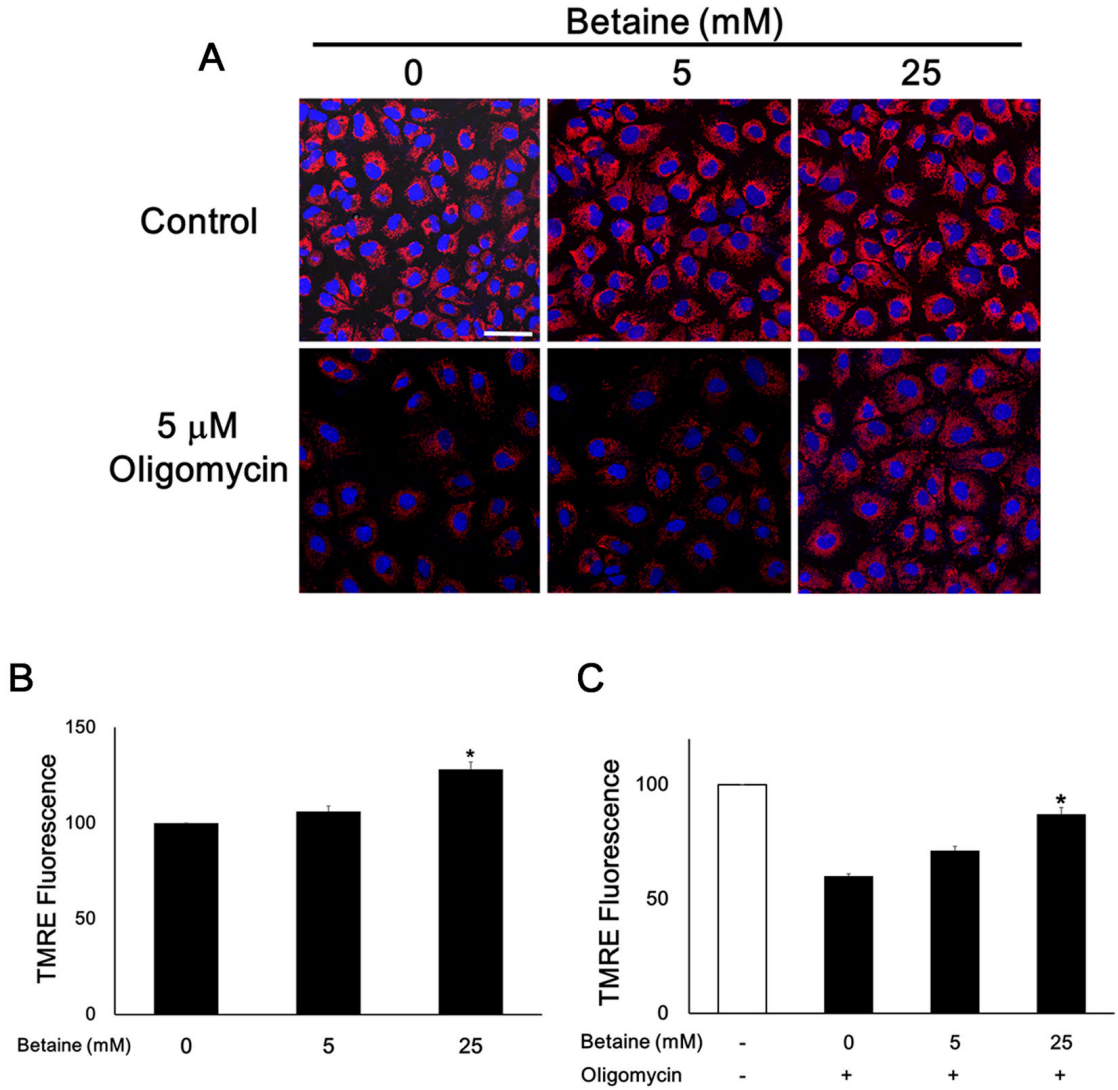
Betaine increases mitochondrial membrane potential. (A) Representative images of mitochondrial staining of TMRE (red for mitochondrial membrane potential. TMRE; blue for nucleus, Hoechst 33342). Scale bar = 40 μm. (B and C) Quantitative analysis of relative intensities of TMRE fluorescence. Data are shown as means ± SEM of measurements taken from each 100 cells from 3 independent experiments. *p < .05 compared to 0 mM betaine (B) and 0 mM betaine with 5 μM oligomycin (C).

To gain insight into whether the mitochondrial elongation morphology induced by betaine affects the ability of mitochondrial function, we examined mitochondrial membrane potential using TMRE (Figure 5). Comparing the basal level of mitochondrial membrane potential without betaine, the intensity of TMRE fluorescence was significantly elevated by betaine in a dose-dependent manner. 25 mM betaine increased the fluorescent intensity about 25%. Next, we tested whether betaine is able to improve the mitochondrial membrane potential of the oligomycin treated cells. Since we did not observe a significant change of membrane potential after the addition of 2 μM oligomycin under our experimental condition, 5 μM oligomycin was used. After pretreatment with 5 μM oligomycin, the remained cells showed almost 50% decrease of mitochondrial membrane potential as shown in Figure 5(C) compared to control cells. Interestingly, mitochondrial membrane potential was returned to 80% of control levels by betaine. The higher dose of betaine (25 mM) seems to have a more positive effect on the membrane potential. These data suggest that betaine can improve the cell survival and membrane potential and protect cells from mitochondrial dysfunction.

### Betaine enhances the mitochondrial fusion in zebrafish

**Figure 6.**
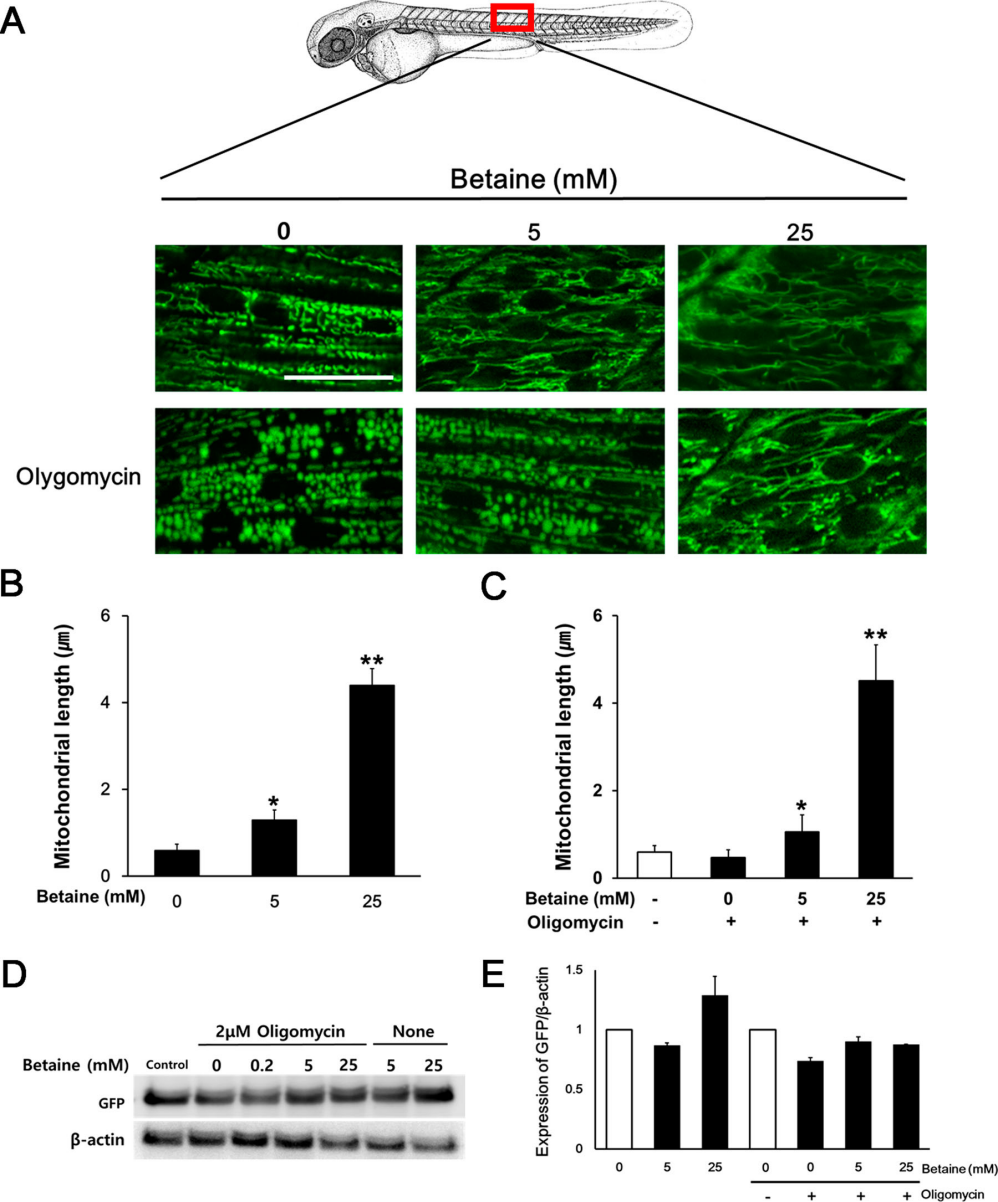
Betaine promotes mitochondrial fusion on the Tg(*mito:*EGFP) zebrafish embryo. (A) Representative images showing the mitochondrial fusion effect of betaine with or without oligomycin treatment on the muscle mitochondria. Scale bar = 20 μm. (B and C) Quantitative analysis of mitochondrial length from betaine or betaine and oligomycin treated embryos. (D and E) Representative image of western blot and quantitative analysis of relative intensity of GFP are presented. Data shown are means ± SEM of measurements taken from 30 embryos from 3 independent experiments. *p < .05, **p < .001 vs 0 mM betaine (B) and 0 mM betaine and 2 μM oligomycin (C).

While we showed that betaine has the ability to extend mitochondrial networks *in vitro,* we investigated if betaine also functions in a similar manner *in vivo*. Since the live monitoring of mitochondrial morphology is not easy in a mammalian system, Tg(*mito*:EGFP) zebrafish was used to observe the change of mitochondrial dynamics for studying mitochondrial physiology and pathogenesis *in vivo* (Kim et al. 2008). Since knockdown of MFN or OPA1 resulted in mitochondrial fragmentation and damaged cristae in drosophila muscle cells (Rana et al. 2017), we visualized the live cell imaging of mitochondrial networks on the muscle cells of Tg(*mito*:EGFP) zebrafish for representing the mitochondrial dynamics. 1 dpf zebrafish embryos were treated with 0, 5, 25 mM betaine for 6 hours. Live cell imaging of the embryos revealed the normal mitochondria morphology and the average length of mitochondria was ∼ 1 μm (Figure 6). Betaine treated embryos had the elongated mitochondria in a concentration dependent manner. In the presence of 25 mM betaine, the length of mitochondria became ∼5-fold longer than control ones.

The zebrafish model system has been characterized to the effect of mitochondrial inhibitors identifying morphological and functional phenotype on zebrafish development and provides a potential *in vivo* model for testing pharmaceutical mitochondria-targeted drugs (Pinho et al. 2013; Byrnes et al. 2018). In particular, oligomycin induced the fragmentation of mitochondria and gastrulation defects to halt the embryogenesis. Therefore we pretreated 1 dpf zebrafish embryos with 2 μM oligomycin for 15 minutes to avoid the early gastrulation arrest. Embryos with oligomycin displayed the short rod-like mitochondria compared to control embryos without developmental defect. After oligomycin pretreatment, embryos were soaked in the 0, 5, 25 mM betaine for 6 hours and lively imaged. The fragmented mitochondria from the oligomycin pretreatment were changed to normal morphology by betaine in a dose-dependent manner and the average length of mitochondria also increased by betaine treatment. In addition, betaine treatment increased the expression of GFP which represents the mass of mitochondria and rescued its mass from oligomycin treatment. Overall data strongly support that betaine enhanced mitochondrial fusion through increasing mitochondrial fusion event from mitochondrial OXPHOS dysfunction.

## Discussion

Mitochondrial dynamics reveal the overall health condition of mitochondria. Healthy mitochondria constantly fuse and divide to keep a dynamic network under physiological conditions (Nunnari and Suomalainen 2012). The morphology of mitochondria is tightly regulated by the balance of fusion and fission events that are mediated by MFN2, DRP1 respectively (Kang et al. 2016). It has shown that the fusion is essential to maintain membrane potential and to keep mitochondrial integrity and function by spreading matrix components and fission is necessary to produce new mitochondria and remove damaged ones from dysfunctional networks (Szabo et al. 2018). Mitochondrial hyperfusion occurs to increase ATP levels to re-optimize mitochondrial function that enables damaged mitochondria to rescue respiratory activity and increases the bioenergetics capacity for cell survival under mild stress conditions like including UV exposure and nutrient deprivation (Legros et al. 2002; Qian et al. 2012).

The dysregulation of mitochondrial fusion and fission is associated with mitochondrial dysfunction and found in several human diseases, such as neurodegenerative diseases like CMT3A, ataxia and Huntington’s disease (Reddy et al. 2011; Godoy et al. 2018). Mitofusins (MFNs) are well-known mitochondrial membrane proteins for mediating mitochondrial fusion. The MEF cells from Mfn1 or Mfn2 mutant mouse displayed fragmented mitochondria and suppression of Mfn2 by antisense nucleotides decreased a mitochondrial network, inhibited the oxidation of glucose and reduced mitochondrial membrane potential in L6E9 myotube cells (Pich et al. 2005). When Mfn2 was overexpressed, mitochondrial membrane potential was increased and the rate of glucose oxidation was enhanced in HeLa cells (Kawalec et al. 2015). On the contrary, Drp1 is a major mitochondrial fission regulator that is closely linked to neurodegenerative diseases (Qian et al. 2012). Dominant-negative Drp1 prevented mitochondrial fragmentation, retained membrane potential and inhibited apoptotic cell death in COS7 cells (Uo et al. 2009).

Our results demonstrated for the first time that betaine itself can promote the mitochondrial fusion and restore fragmented into elongated and tubular mitochondria over mitochondrial OXPHOS dysfunction induced by oligomycin and rotenone. This enhancing fusion activity of betaine helped to improve mitochondrial function and increase the chance of the survival (Figure 4). Intriguingly, we further discovered that betaine regulates mitochondrial dynamics through a fission-fusion mechanism by stimulating levels of MFN2 and decreasing levels of DRP-1 in a dose-dependent manner. The effects of betaine on MFN2 and DRP1 regulation might provide a way to restore mitochondrial function from OXPHOS disruption.

It was suggested that betaine enhances AKT pathway and decreases AMPK activation (Apicella et al. 2013). Akt is known to stimulate mitochondrial biogenesis, OXPHOS and ATP production (Parra et al. 2014). It was shown that stimulation of mitochondrial fusion by insulin is controlled by Opa-1 that is mediated by Akt-mTOR-NFκB signaling in cardiomyocytes (Parra et al. 2014). On the other hand, AMPK induced mitochondrial fragmentation mediated by Drp1 in human U2OS cells and phosphorylation of AMPK promoted mitochondrial fission (Zhang and Lin 2016). Therefore it is possible that enhanced mitochondrial fusion, improved mitochondrial potential and cell survival by betaine might be mediated by activation of AKT signaling or downregulation of AMPK signaling. More study is needed to define the molecular signaling mechanisms of fusion/fission by of betaine.

In conclusion, our results indicate that betaine can regulate mitochondrial dynamics through fission and fusion events and betaine-mediated mitochondrial changes positively reverse from mitochondrial OXPHOS dysfunction to normal physiological status. Therefore, discovery for new compounds like betaine having enhancing ability of mitochondrial fusion, DRP1 inhibitors, or Mfn2 agonists are of great interest for the therapeutic potential for the treatment of mitochondrial dysfunction-related diseases.
